# Across the Border: A Metamizole Drug-Induced Liver Injury

**DOI:** 10.7759/cureus.81010

**Published:** 2025-03-22

**Authors:** Swati Mahapatra, Shivangini Duggal, Monica Botros, Jesus Guzma, Nawar Hakim, Ricardo Badillo

**Affiliations:** 1 Internal Medicine, Texas Tech University Health Sciences Center El Paso, El Paso, USA; 2 Pathology, Texas Tech University Health Sciences Center El Paso, El Paso, USA; 3 Gastroenterology, Texas Tech University Health Sciences Center El Paso, El Paso, USA

**Keywords:** analgesic combination, dipyrone, drug-induced liver injury, liver failure, metamizole

## Abstract

Metamizole (dipyrone) is a medication with analgesic, antipyretic, and spasmolytic properties, widely used in Mexico and other Latin American countries. It is often grouped with non-steroidal anti-inflammatory drugs (NSAIDs) due to its pain-relieving and fever-reducing effects, making it a common over-the-counter option. However, concerns over its potential to cause agranulocytosis led to its removal from the U.S. market. Reports of drug-induced liver injury (DILI) related to metamizole are uncommon but noteworthy. Given its frequent use in Latin America and parts of Europe, awareness of this possible adverse effect is important.

A 39-year-old man with no prior medical conditions sought medical care for persistent right upper quadrant pain and jaundice lasting two weeks. To address his symptoms, he had been prescribed a compounded medication containing butylscopolamine and metamizole in Ciudad Juárez, Mexico. Shortly after, he noticed yellowing of his skin and eyes, prompting him to seek care in the United States. Laboratory findings indicated a mixed hepatocellular pattern of liver injury (total bilirubin: 6.5, direct bilirubin: 5.1, aspartate aminotransferase: 1,496, alkaline phosphatase: 130, and alanine aminotransferase: 2,579). Additional testing showed elevated transferrin saturation and ferritin levels (82%; 57,700). Imaging of the abdomen revealed periportal edema without other significant abnormalities. A thorough evaluation for viral, autoimmune, and genetic liver diseases yielded negative results. A liver biopsy demonstrated features of portal and lobular hepatitis with eosinophilic infiltration, suggesting DILI. Prussian blue staining did not indicate iron accumulation. Given the timing and exclusion of other causes, the reaction was attributed to the metamizole-containing medication. Discontinuation of the drug and supportive care led to biochemical improvement and resolution of jaundice.

Metamizole-related DILI may be underrecognized, particularly in regions where the drug is no longer available. However, it remains widely used in Latin America, relevant for clinicians treating immigrant populations. Recognizing international medication use and obtaining a thorough drug history is essential, especially when managing patients exposed to pharmaceuticals not routinely encountered in the United States.

## Introduction

Metamizole (dipyrone) is an analgesic, antipyretic, and spasmolytic agent commonly used in Mexico. It is most frequently classified as a non-steroidal anti-inflammatory drug (NSAID), and its antipyretic effect has made it a popular over-the-counter home medication in Latin American countries [1]. However, metamizole has not been available in the United States since 1977 due to its well-established risk of agranulocytosis [1]. Additionally, drug-induced liver injury (DILI) has also been described in the literature as a notable adverse effect of the medication. Given metamizole’s widespread use in Latin America and European countries and its ease of access in U.S. border communities, we aim to highlight the potential risks associated with its use. Below, we present a case of DILI linked to metamizole use and a discussion of the existing literature on the topic.

This case was previously presented as a meeting abstract at the 2024 Digestive Diseases Week Annual Meeting on May 20, 2024.

## Case presentation

Metamizole (dipyrone), once widely used as an analgesic and antipyretic agent, has been associated with rare cases of DILI. We present the case of a 39-year-old Hispanic male who developed severe right upper quadrant pain and jaundice after ingesting a compounded medication containing metamizole in Ciudad Juárez, Mexico. He sought medical evaluation across the border.

**Figure 1:**
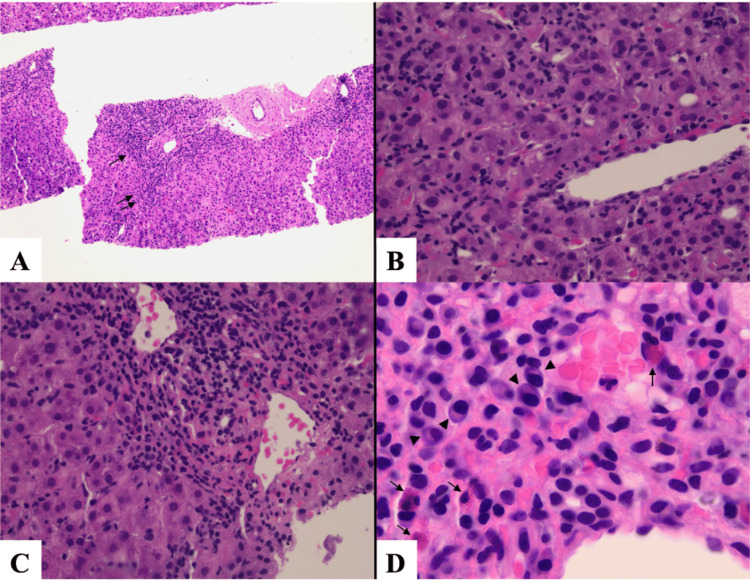
Pathology images A: Low power view shows the involvement of the portal tracts and hepatic lobules with this inflammatory process (H&E stain, original magnification x100). B: Lobular activity characterized by leukocytes infiltrating among hepatocytes (H&E stain, original magnification x400). C: Portal tract expanded by inflammation (H&E stain, original magnification x400). The inflammatory cells consist of lymphocytes, eosinophils (single arrows), plasma cells (arrowheads), rare neutrophils, and other mononuclear leukocytes (H&E stain, original magnification x1000, oil immersion). H&E: hematoxylin and eosin

On admission, laboratory tests revealed a hepatocellular pattern of liver injury, with marked elevations in transaminases and evidence of iron overload (Table 1). Imaging showed periportal edema in the liver with associated gallbladder wall thickening, likely related to acute hepatitis. A subsequent workup for viral, autoimmune, and hereditary causes of liver injury was unremarkable. A liver biopsy confirmed hepatic parenchymal and lobular hepatitis with a prominent eosinophilic infiltrate, findings consistent with DILI attributed to metamizole (Figure 1).

**Table 1:** Laboratory values on admission AST: aspartate aminotransferase, GOT: glutamate-oxaloacetate transaminase, ALT: alanine aminotransferase, GPT: glutamate-pyruvate transaminase, IgM: immunoglobulin M, INR: international normalized ratio

Tests	Results	Normal range
White blood count	5.46 x 103/uL	4.50-11.03/uL
Hemoglobin	17.2 g/dL H	12.0-15.0 g/dL
Platelets	150 x 103/uL	150-450 x 103/uL
Sodium, serum	137 mmol/L	135-145 mmol/L
Creatinine	0.90 mg/dL	0.70-1.3 mg/dL
Albumin	3.9 g/dL	3.4-5.4 g/dL
Total bilirubin	6.5 mg/dL H	0.1-1.2 mg/dL
Bilirubin direct	5.10 mg/dL H	<0.3 mg/dL
AST (GOT)	1496 IU/L H	14-20 IU/L
ALT (GPT)	2579 IU/L H	29-33 IU/L
Alkaline phosphatase	130 IU/L	44-147 IU/L
Iron serum	306 mcg/dL H	60-170 mcg/dL
% transferrin saturation	66% H	15-50%
Ferritin	5770 ng/mL H	12-300 ng/mL
Hepatitis B surface antigen	Negative	Negative
Hepatitis B core antigen	Negative	Negative
Hepatitis A IgM	Negative	Negative
Hepatitis C	Negative	Negative
Antinuclear antibody	Negative	Negative
INR	1.2	0.9-1.1

Management involved discontinuing the medication and providing supportive care, which led to significant clinical improvement. This case highlights the underrecognized risk of metamizole-induced DILI, particularly in regions where the drug remains accessible despite regulatory restrictions in other countries. Clinicians practicing in areas with significant cross-border healthcare interactions should maintain a high index of suspicion for medications not approved locally. This underscores the importance of obtaining comprehensive medication histories in patients presenting with acute liver injury.

## Discussion

DILI is a common condition in which medications, whether prescribed or over-the-counter, cause hepatocyte and biliary/portal triad inflammation [2]. Drug toxicity can occur via one of two mechanisms: the intrinsic mechanism, in which damage is directly caused by the medication and is dose-dependent, or the idiosyncratic mechanism, in which the degree of damage is influenced by environmental and host interactions with the medication [2]. A definitive diagnosis of DILI is established using a causality assessment tool such as the Roussel Uclaf Causality Assessment Method score [3].

Metamizole (dipyrone) is an analgesic, antipyretic, and spasmolytic agent commonly used in Mexico, South America, Germany, and Russia. It is most frequently classified as an NSAID [1,2]. However, in the United Kingdom, the United States, and some Scandinavian countries, metamizole has been withdrawn from the market due to serious side effects, the most severe being agranulocytosis.

A growing body of literature has also identified metamizole as a rare cause of idiosyncratic liver injury, further emphasizing the importance of obtaining a thorough medication history in patients presenting with acute liver injury, particularly in regions where this medication is available [4,5]. The presentation of metamizole-induced DILI varies among patients; however, prior studies suggest that up to 66% of affected patients develop jaundice with peak bilirubin levels exceeding 3 mg/dL, and up to 22% of patients with prolonged metamizole use may present with acute liver failure [6].

Metamizole is a prodrug converted to N-methyl-4-aminoantipyrine (MAA) via extensive hepatic metabolism utilizing the CYP450 enzyme system [5,7]. MAA is largely responsible for the medication’s analgesic effect and reaches a maximum serum concentration of 10 to 20 µg/mL after 1 g of metamizole [3]. MAA then undergoes N-demethylation and is converted to 4-aminoantipyrine (AA), which is subsequently formylated to N-formyl-4-aminoantipyrine (FAA).

Prior studies have helped elucidate the specific etiologies of DILI, further characterize the injury pattern, and highlight additional demographic and disease associations. In 2020, Sebode et al. performed a retrospective analysis of 154 cases of DILI and their etiologies. Of these cases, 14.9% were attributed to metamizole use, with the dominant injury pattern being hepatocellular. This pattern was characterized by alanine aminotransferase levels exceeding aspartate aminotransferase levels, similar to the findings in our patient [1]. Furthermore, prior literature has shown that liver biopsy's most common histopathological finding is an inflammatory infiltrate with prominent eosinophilic cells [8].

In 2021, Weber et al. conducted a subgroup analysis of 32 patients with metamizole-associated DILI among 238 DILI cases. This analysis revealed that suspected metamizole-associated DILI was associated with female sex, a high proportion of antinuclear antibody positivity, a hepatocellular pattern of injury, and a predominance of eosinophilic cell infiltration and necrosis on histopathological analysis [7]. Additionally, Reike-Kunze et al. performed a retrospective analysis of 157 severe DILI cases presented to their center in Hamburg, Germany, between 2008 and 2018. In this study, 13% of patients were found to have DILI secondary to metamizole use. The authors reported a latency period of 28 days between the first drug intake and the initial clinical presentation of liver injury. Moreover, they found that the presence of confluent necrosis of any grade on liver histology was associated with poor outcomes, including liver failure, sepsis, and intracerebral hemorrhage [8].

In addition to being an independent risk factor for DILI, metamizole has been shown to have a stronger association with liver injury than other common analgesic/antipyretic medications. In a 2021 retrospective cohort analysis, Hedenmalm et al. compared the risk of liver injury in patients receiving either metamizole or paracetamol (acetaminophen) using data from the Intercontinental Medical Statistics Disease Analyzer Germany database, selecting patients who met the relevant inclusion criteria. They found that metamizole was associated with a higher risk of liver injury compared to paracetamol (adjusted hazard ratio: 1.69, 95% confidence interval: 1.46-1.97). No association was found between liver injury and sex, naproxen use, or alcohol use in either group [9].

Our patient presented with evidence of acute liver injury but without signs of overt acute liver failure and had a Model for End-Stage Liver Disease score of 17. He sought medical attention at our institution within 14 days of starting metamizole. His pathology did not reveal necrosis, and with conservative management, he recovered well upon discontinuation of the medication.

## Conclusions

Metamizole is a commonly used analgesic in Latin America and Europe. However, it has been discontinued in the United States and the United Kingdom due to the risk of agranulocytosis. DILI is a known but often overlooked complication of metamizole use. As clinicians, particularly those practicing along the United States-Mexico border and in other areas with high immigration, it is crucial to recognize the use of internationally available medications and obtain a thorough medical history from patients.
